# Predicting protein-protein interactions using high-quality non-interacting pairs

**DOI:** 10.1186/s12859-018-2525-3

**Published:** 2018-12-31

**Authors:** Long Zhang, Guoxian Yu, Maozu Guo, Jun Wang

**Affiliations:** 1 College of Computer and Information Sciences, Southwest University, Chongqing, China; 2 School of Electrical and Information Engineering, Beijing University of Civil Engineering and Architecture, Beijing, China; 3 Beijing Key Laboratory of Intelligent Processing for Building Big Data, Beijing, China

**Keywords:** Protein-protein interactions, Non-interacting proteins, Deep neural networks, Sequence similarity, Random walk

## Abstract

**Background:**

Identifying protein-protein interactions (PPIs) is of paramount importance for understanding cellular processes. Machine learning-based approaches have been developed to predict PPIs, but the effectiveness of these approaches is unsatisfactory. One major reason is that they randomly choose non-interacting protein pairs (negative samples) or heuristically select non-interacting pairs with low quality.

**Results:**

To boost the effectiveness of predicting PPIs, we propose two novel approaches (NIP-SS and NIP-RW) to generate high quality non-interacting pairs based on sequence similarity and random walk, respectively. Specifically, the known PPIs collected from public databases are used to generate the positive samples. NIP-SS then selects the top-*m* dissimilar protein pairs as negative examples and controls the degree distribution of selected proteins to construct the negative dataset. NIP-RW performs random walk on the PPI network to update the adjacency matrix of the network, and then selects protein pairs not connected in the updated network as negative samples. Next, we use auto covariance (AC) descriptor to encode the feature information of amino acid sequences. After that, we employ deep neural networks (DNNs) to predict PPIs based on extracted features, positive and negative examples. Extensive experiments show that NIP-SS and NIP-RW can generate negative samples with higher quality than existing strategies and thus enable more accurate prediction.

**Conclusions:**

The experimental results prove that negative datasets constructed by NIP-SS and NIP-RW can reduce the bias and have good generalization ability. NIP-SS and NIP-RW can be used as a plugin to boost the effectiveness of PPIs prediction. Codes and datasets are available at http://mlda.swu.edu.cn/codes.php?name=NIP.

## Background

As the essential component of all organisms, proteins form the very basis of life and carry out a variety of biological functions within living organisms. A protein rarely accomplishes its functions alone, instead it interacts with other proteins to accomplish biological functions. It is thus generally accepted that protein-protein interactions (PPIs) are responsible for most activities of living organisms. As a hotspot of proteomics research, detecting PPIs can not only provide great insight for understanding various biological functions in cells, but also contribute to develop drugs for special diseases[1, 2]. In the past decades, different high-throughput technologies had developed to detect PPIs, such as tandem affinity purification (TAP) [3], co-immunoprecipitation (Co-IP) [4], x-ray crystallography [5], yeast two-hybrid (Y2H) screens [6, 7], and mass spectrometric protein complex identification (MS-PCI) [8]. However, these wet-experiment based solutions are costly and tedious. PPIs obtained from these biological experiments only cover a small fraction of the complete PPI network [9]. Furthermore, these high-throughput technologies generally suffer from high rates of false negatives and false positives [9–11].

Computational approaches have been developed to predict PPIs in an economic and reliable way. These approaches use different data types to predict PPIs, such as protein domains [12], protein structure information [13], gene neighborhood [14], gene fusion [15], and phylogenetic profiles [16, 17]. Nevertheless, these methods are barely achieved if the pre-knowledge of the proteins is not available, i.e., protein functional domains, 3D structure of proteins, and other information [18]. As the explosive growth of sequence data, more and more researchers have moved toward sequence data based approaches to predict PPIs. Experimental results show that it is adequate to predict new PPIs using amino acid sequences alone [19–27].

Martin et al. [19] extracted the feature information of amino acid sequences by the extended signature descriptor and used support vector machine (SVM) to predict PPIs [19]. Shen et al. [20] adopted SVM as the classifier and encoded the feature information of amino acid sequences by conjoint triad (CT), in which the 20 standard amino acids are grouped into 7 categories on the basis of their dipoles and volumes of the side chains. This SVM-based approach yields a high prediction accuracy of 83.9%. However, this approach can not sufficiently encode the feature information, since CT only takes into account the neighboring effect of amino acid sequences, but PPIs usually occur at the non-continuous segments of amino acid sequences. Guo et al. [21] employed the auto covariance (AC) to detect the correlation among discontinuous segments and obtained an accuracy of 86.55%. You et al. [24] combined a novel multi-scale continuous and discontinuous (MCD) feature representation and SVM to predict PPIs. MCD feature representation can adequately capture continuous and discontinuous feature information of segments within an amino acid sequence. This method yields a high accuracy of 91.36% [24]. Different from these SVM-based approaches, Yang et al. [22] combined *k*NN and local descriptor (LD) to predict PPIs and obtained an accuracy of 83.73%. Du et al. [27] applied deep neural networks (DNNs) and integrated diverse feature descriptors to encode the feature information of amino acid sequences to predict PPIs. This approach obtains a high accuracy of 92.5% on predicting PPIs of *Saccharomyces cerevisiae* [27]. Wang et al. [28] used DNNs and a novel feature descriptor named local conjoint triad descriptor (LCTD), which encodes continuous and discontinuous feature information of local segments within an amino acid sequence, to predict PPIs. This approach yields a high accuracy of 93.12% on PPIs of *Saccharomyces cerevisiae*.

However, the performance of all the aforementioned sequence-based methods heavily depend on the quality of PPIs datasets. Positive examples (interacting protein pairs) are generally chosen based on reliable methods (small scale experiments), interactions confirmed by Y2H [6, 7], Co-IP [4], and other methods; or interactions confirmed by interacting paralogs [29, 30]. Therefore, given the public protein-protein interactions databases [31], the positive examples are readily available and can be easily constructed. The *difficulty* is that there are no ‘gold standard’ of non-interacting protein pairs (negative examples), which contribute to discriminatively predict PPIs. Two kinds of strategies are widely used by previous computational methods [19–21, 23–27]. The first one randomly pairs proteins and then removes the pairs included in the positive examples [21, 30]. The second constructs negative examples based on the subcellular localization of proteins [23, 25–27]. However, these two strategies have limitations and may compromise the prediction performance. The first strategy wrongly takes a large number of positive samples as negative samples, while the second strategy leads to a biased estimation of PPIs prediction [30].

In this paper, two novel approaches (NIP-SS and NIP-RW) are proposed to improve the performance of PPIs prediction. NIP-SS and NIP-RW separately generate reliable non-interacting pairs (negative dataset) based on sequence similarity and on random walk in the PPIs network. The basic idea of NIP-SS is: given a positive protein pair (*i* and *j*), and a protein *k*, the larger the sequence difference between *i* and *k* is, the smaller the probability that *k* interacts with *j* (*i*) is. In addition, we control the degree distribution of selected protein pairs to make it similar as that of the positive dataset. Given a PPI network $\mathcal {G} =(\mathcal {V},\mathcal {E})$, where $\mathcal {V}$ is the set of proteins, and $\mathcal {E}$ is the set of weighted undirected edges, where the weight reflects the interaction strength between a protein pair, 1 means an interaction, 0 means unknown. The basic idea of NIP-RW is: after a *k*-steps random walk on $\mathcal {G}$, if the edge weight between two proteins is larger than 0, there may be an interaction between them; otherwise, there may be no interaction.

To investigate the effectiveness of NIP-SS and NIP-RW, we firstly collected the positive sets from Database of Interacting Proteins (DIP) [31], and separately constructed negative sets using four strategies : 1) NIP-SS, 2) NIP-RW, 3) subcellular localization, 4) random pairing, and then merged the positive set and each negative set to form a training dataset. Next, we used the auto covariance (AC) [21] descriptor to extract the features from amino acid sequences and Deep neural networks (DNNs) to predict PPIs. AC can account for the interactions between residues with a certain distance apart in the sequence and encode the features by a lower dimensional vector [21], DNNs can automatically extract high-level abstractions and reduce the model training time [32]. We performed comparative and quantitative experiments on public benchmark datasets to study the effectiveness of negative datasets generated by different strategies. The experimental results show that NIPI-SS and NIP-RW have good generalization ability and contribute to a higher accuracy in predicting PPIs than other related and widely-used strategies.

## Methods

### PPIs datasets

To comprehensively evaluate the rationality of NIP-SS and NIP-RW, we constructed 3 non-redundant positive PPIs sets for *S. cerevisiae*, *H. sapiens*, and *M. musculus* from DIP [31]. Next, we separately generated negative PPIs (non-interacting protein pairs) for these three species using NIP-SS, NIP-RW, subcellular location, and random pairing. After that, we merged the positive and negative sets for each species. As a result, twelve PPIs datasets are obtained. In addition, another six datasets were collected as the independent test datasets to further assess the generalization ability of NIP-SS and NIP-RW, *Mammalian* dataset collected from Negatome 2.0 [33] only contains non-interacting protein pairs, they were generated by manual curation of literature. The procedure of constructing the negative dataset will be introduced later.

The twelve datasets are divided into three groups based on the species. The experimental-validated PPIs of these three groups are all from DIP [31]. The first group contains 17257 positive PPIs of *S. cerevisiae* (version 20160731) collected by Du et al. [27]. The second and third groups are processed by ourselves, they contain 3355 and 923 positive PPIs of *H. sapiens* and *M. musculus*(version 20170205), respectively. These positive PPIs are generated by excluding proteins with fewer than 50 amino acids and with ≥ 40% sequence identity by cluster analysis via the CD-HIT program [34]. The excluded proteins have a heavy impact on the performance of PPIs prediction [21]. Each of these three groups contains four training sets and the difference between these four sets is the negative samples, which are generated by NIP-SS, NIP-RW, subcellular location, and random pairing. Table 1 gives the statistics of these 18 datasets.

**Table 1:** The 18 PPIs datasets used in this paper

Groups	Datasets	# Positive samples	# Negative samples
*SC* ^a^	*SC-SS* ^1^	17257	17257
	*SC-RW* ^2^	17257	17257
	*SC-Sub* ^3^	17257	17257
	*SC-RP* ^4^	17257	17257
*HS* ^b^	*HS-SS*	3355	3355
	*HS-RW*	3355	3355
	*HS-Sub*	3355	3355
	*HS-RP*	3355	3355
*MM* ^c^	*MM-SS*	923	923
	*MM-RW*	923	923
	*MM-Sub*	923	923
	*MM-RP*	923	923
*Test* ^d^	*C. elegans*	4013	0
	*E. coli*	6984	0
	*H. sapiens*	1412	0
	*H. pylori*	1420	0
	*M. musculus*	313	0
	*Mammalian*	0	1937

^a^*SC: S. cerevisiae*;

^b^*HS: H. sapiens*;

^c^*MM: M. musculus*;

^d^*Test*: Six independent testing datasets;

^1^NIP-SS;

^2^NIP-RW;

^3^Subcellular location;

^4^Random pairing

#### Generating non-interacting protein pairs

Negative samples must be chosen with caution, which can heavily affect the performance of PPIs prediction. There are two primary strategies to construct negative samples, including random pairing and subcellular location. For the *first* strategy, after constructing the positive set from DIP, we count the number of proteins in the positive set and put these proteins into set $\mathcal {P}$. Next, we can randomly select two proteins from $\mathcal {P}$ and take them as a non-interacting pair if they do not have an interaction in the positive set. Obviously, this random pairing is not completely reliable, it will produce a high rate of false negatives for generated negative examples, since the interactions between proteins in the DIP are far from complete.

The *second* strategy is based on a hypothesis that proteins located in different subcellular localizations do not interact. A protein can be divided into seven groups based on subcellular location information extracted from Swiss-Prot (http://www.expasy.org/sprot/), including cytoplasm, nucleus, mitochondrion, endoplasmic reticulum, golgi apparatus, peroxisome and vacuole. The negative samples are obtained by pairing a protein from one group with another protein from the other groups. These negative samples further exclude the proteins pairs appeared in the positive set. However, Ben-Hur and Noble [30] proved that subcellular localization based approaches lead to a biased accuracy of PPIs prediction.

Motivated by the limitations of existing solutions, we proposed two novel approaches NIP-SS and NIP-RW to construct the negative datasets. Let $\mathcal {G} = (\mathcal {V}, \mathcal {E})$ encode a PPIs network, where $\mathcal {V}$ is the set of proteins, and $\mathcal {E}$ stores the known interactions. To construct a reliable negative dataset with good generalization ability, we hope that proteins in the negative dataset are as many as possible. The average repeatability can be employed to describe the generalization ability of a dataset, which is calculated by $r = \sum _{i=1}^{n}(d(i)-1)/n$, where *d*(*i*) means the degree of protein *i*. Note, if a protein in the negative dataset does not ‘interact’ with five proteins, then this protein have a degree of five. The smaller the value of *r*, the larger the generalization ability of this dataset is. On the one hand, we also hope that the degrees of proteins in the negative dataset are not too small, proteins with low degrees contain little predictive information and are not conducive for predicting PPIs. On the other hand, the degrees of proteins should not be too large, which will lead to an overestimation of prediction results. In addition, the maximum degree of proteins, the proportion of proteins in different ranges of degrees, and the proportion of non-interactions in each range all have an impact on the prediction performance. Given these reasons, we need to construct a reliable negative dataset, in which the degree distribution of proteins and interaction distribution are similar to those in the positive dataset. Such a negative dataset contains more proteins and has less bias.

#### Generating non-interacting protein pairs based on sequence similarity

The basic idea of NIP-SS is that, for an experimental validated PPI between protein *i* and *j*, if a protein *k* is dissimilar to *i*, there is a low possibility that *k* interacts with *j*. Based on this idea, we firstly generate the positive set of proteins $\mathcal {P}$ having confirmed interactions between another protein, and compute the sequential similarity between any two proteins in $\mathcal {P}$. Next, we sort the sequence similarity between all protein pairs in $\mathcal {P}$ by the ascending order, and then select the top-*m* protein pairs with the lowest similarity as negative examples (non-interacting pairs), *m* is generally larger than the number of positive examples to facilitate the follow-up adjustment. If we employ these negative examples to form a negative dataset and then use this dataset to predict PPIs, it will lead to an over-estimation of PPIs prediction. This is because such negative dataset contains some proteins with very large degrees, which occur more frequently in the negative dataset than in the positive dataset. For example, the maximum degree in the positive dataset is 252, but 1439 in the initial negative dataset (see “Contribution of controlling degrees” section). As such, the bias is introduced into the training set composed with positive samples and negative samples. To ensure a good generalization ability, the degree distribution of proteins needs to be controlled during constructing the negative dataset.

**Fig. 1:**
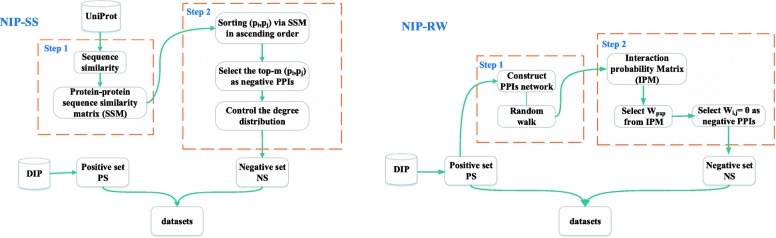
The flowchart of constructing reliable negative samples. The left and right of this Figure describe the strategy of NIP-SS and NIP-RW, respectively

We advocate to make the degree distribution of proteins in the negative dataset similar with that of the positive dataset. We firstly calculate the degree distribution of proteins, maximum degree, the proportion of proteins and the number of interactions in different ranges of degrees (such as the degree ≤ 10, the degree in (11,20], and so on) in the positive dataset. Similarly, we also compute the above values in the negative dataset. Next, we compare these values of positive and negative datasets, and then adjust the number of non-interacting partners of a protein by referring to the corresponding values of the positive dataset. Finally, we remove the protein pairs appeared in the positive dataset to generate the reliable negative dataset. The process of NIP-SS is shown in Fig. 1.

We collect the amino acid sequences data from the UniProt database [35]. Sequence similarity between two proteins *i* and *j* is calculated using blocks substitution matrix (BLOSUM), which is a substitution matrix used for sequence alignment of proteins [36]. BLOSUM matrices are used to score alignments between evolutionary divergent protein sequences. We adopt BLOSUM50 to compute the score between proteins, and then normalize the score as follows:

1 $$ \tilde{bl}(i,j) = \frac{bl(i,j) - min\{bl(i,1),\cdots, bl(i,n)\}}{max\{bl(i,1), \cdots, bl(i,n)\}}  $$

where *n* is the total number of proteins in $\mathcal {P}$, *bl*(*i*,*j*) is the original BLOSUM50 score of protein *i* and *j*.

#### Generating non-interacting pairs based on random walk

NIP-RW is motivated by the observation that interacting proteins are likely to share similar functions, level-1 (*k*=1) neighborhood (or directly interacting) proteins are more probable to share functions than level-2 (*k*=2) neighborhood proteins, whose interactions are mediated by another protein. In other words, the probability of sharing similar functions reduces as the increase of *k* [37]. Given that, two proteins that can only be connected after a *k*-step random walk, is less likely to share functions and thus less probable to interact with each other. The flowchart of NIP-RW is shown in Fig. 1.

Let $\mathcal {G} = (\mathcal {V}, \mathcal {E})$ represent a PPI network, where $\mathcal {V}$ is the set of proteins, and $\mathcal {E}$ is the set of edges. Each vertex $u\in \mathcal {V}$ stands for a unique protein, each edge $(u,v)\in \mathcal {E}$ represents an observed interaction between protein *u* and protein *v*, $\mathbf {E}\in \mathbb {R}^{n \times n}$ stores available interactions between *n* proteins. We define a pair of proteins (*u* and *v*) as level-*k* neighbors if there exists a path *ϕ*=(*u*,⋯,*v*) with length *k* in $\mathcal {G}$. The *k*-steps random walk process can be modeled as follows:

2 $$ \mathbf{W}^{(k)} = \mathbf{W}^{(k-1)}\mathbf{E}  $$

After *k*-steps random walk, we can obtain a updated adjancency matrix $\mathbf {W}^{(k)} \in \mathbb {R}^{n \times n}$, which reflects the inferred interaction probability (strength) between any pairwise proteins.

Since **E** is generally very sparse, **W**^(*k*)^ still encodes a sparse matrix. As such, the selected negative examples are inclined to proteins connected with few proteins, and lead to a bias of negative examples. To generate a negative dataset with good generalization ability, we use a sub-matrix **W**_*p*×*p*_ of **W**^(*k*)^ to control the number of proteins and degree distribution of these selected *p* proteins. After that, we select two proteins with **W**_*p*×*p*_(*i*,*j*)=0 and take these two proteins as a non-interacting pair. We will investigate the parameter sensitivity of *p* and provide a principal way to specify *p* in “Contribution of controlling degrees” section.

### Feature vector extraction

To effectively predict PPIs based on amino acid sequences, we need to extract and represent the essential information of interacting proteins by a feature descriptor. Many feature descriptors have been utilized to predict PPIs. Among these descriptors, conjoint triad (CT) [20] only takes into account the neighboring effect of amino acid sequences. However, PPIs generally occur at discontinuous segments of amino acid sequences. Local descriptor (LD) [23], auto covariance (AC) [21], multi-scale continuous and discontinuous (MCD) [24] and local conjoint triad descriptor (LCTD) [28] can effectively address this problem and achieve better prediction. Among these four descriptors, feature vectors encoded by AC have the lowest dimensionality. To balance the effectiveness and efficiency, we employ AC to encode the feature information of amino acid sequences, and then use DNNs to predict PPIs. To be self-inclusive, we introduce the AC feature descriptor in the following subsection.

#### Auto covariance (AC)

PPIs generally can be divided into four interaction modes: electrostatic, hydrophobic, hydrogen bond, and steric [38]. Seven physicochemical properties of amino acids can reflect these interaction modes whenever possible, including hydrophobicity [39], hydrophilicity [40], volumes of side chains of amino acids [41], polarity [42], polarizability [43], solvent-accessible surface area [44], net charge index of side chains [45]. The original values of these seven physicochemical properties for each amino acid are shown in Table 2. Feature normalization can improve the accuracy and efficiency of mining algorithms on the data [46]. Given that, we firstly normalize data with zero mean and unit standard deviation as follows:

3 $$ P'_{ij} = \frac{P_{i,j}- \tilde{P_{j}}}{S_{j}}  $$

**Table 2:** The original values of the seven physicochemical properties for each amino acid

Code	*H*_1_	*H*_2_	*V*	*P*_1_	*P*_2_	*SASA*	*NCI*
A	0.62	-0.5	27.5	8.1	0.046	1.181	0.007187
C	0.29	-1	44.6	5.5	0.128	1.461	-0.03661
D	-0.9	3	40	13	0.105	1.587	-0.02382
E	-0.74	3	62	12.3	0.151	1.862	0.006802
F	1.19	-2.5	115.5	5.2	0.29	2.228	0.037552
G	0.48	0	0	9	0	0.881	0.179052
H	-0.4	-0.5	79	10.4	0.23	2.025	-0.01069
I	1.38	-1.8	93.5	5.2	0.186	1.81	0.021631
K	-1.5	3	100	11.3	0.219	2.258	0.017708
L	1.06	-1.8	93.5	4.9	0.186	1.931	0.051672
M	0.64	-1.3	94.1	5.7	0.221	2.034	0.002683
N	-0.78	2	58.7	11.6	0.134	1.655	0.005392
P	0.12	0	41.9	8	0.131	1.468	0.239531
Q	-0.85	0.2	80.7	10.5	0.18	1.932	0.049211
R	-2.53	3	105	10.5	0.291	2.56	0.043587
S	-0.18	0.3	29.3	9.2	0.062	1.298	0.004627
T	-0.05	-0.4	51.3	8.6	0.108	1.525	0.003352
V	1.08	-1.5	71.5	5.9	0.14	1.645	0.057004
W	0.81	-3.4	145.5	5.4	0.409	2.663	0.037977
Y	0.26	-2.3	117.3	6.2	0.298	2.368	0.023599

*H*_1_: hydrophobicity; *H*_2_: hydrophilicity; *V*: volume of side chains; *P*_1_: polarity; *P*_2_: polarizability; *SASA*: solvent accessible surface area; *NCI*: net charge index of side chains

where *P*_*i*,*j*_ is the *j*-th physicochemical property value for the *i*-th amino acid, $\tilde {P_{j}}$ is the mean of the *j*-th physicochemical property over 20 amino acids and *S*_*j*_ is the corresponding standard deviation of the *j*-th physicochemical property. Then each amino acid sequence is translated into seven vectors with each amino acid represented by the normalized values.

AC is a statistical tool introduced by Wold et al. [38], it is adopted to transform amino acid sequences into uniform matrices. AC can account for the interactions between residues using a certain *lag* apart the entire sequence. To represent an amino acid sequence *A* with length *l*, the AC variables are computed as:

4 $$\begin{array}{*{20}l} AC(lag,j) =& \frac{1}{l-lag}\sum_{i=1}^{l-lag}\left(A_{ij}-\frac{1}{l}\sum_{i=1}^{l} A_{i,j}\right)\\ &\times\left(A_{(i+lag),j}-\frac{1}{l}\sum_{i=1}^{l}A_{i,j}\right) \end{array} $$

*lag* is the distance between residues. *A*_*ij*_ is the *j*-th physicochemical property of the *i*-th amino acid of *A*, *l* is the length of the amino acid sequence *A*. In this way, the number of AC variables is *D*=*lg*×*p*, where *p* is the number of descriptors, which is set as 7 according to seven properties of amino acids. *lg* is the maximum distance *lag*(*lag*=1,2,...,*lg*), which is set as 30 [21]. After that, each amino acid sequence is encoded by a 210-dimensional vector with AC variables. Finally, feature vectors of two individual proteins are taken as inputs of two separate DNNs, respectively.

### Deep neural networks

Deep learning, the most active field in machine learning, attempts to learn multi-layered models of inputs. It has been achieving great success in many research areas, such as speech recognition [47], signal recognition [48], computer vision [49–51], natural language processing [52, 53] and so on. Meanwhile, it also has been widely employed in bioinformatics [54, 55]. Deep learning is not only good at automatically learning the high-level features from the original data, but also good at discovering intricate structures in high-dimensional data [56].

**Fig. 2:**
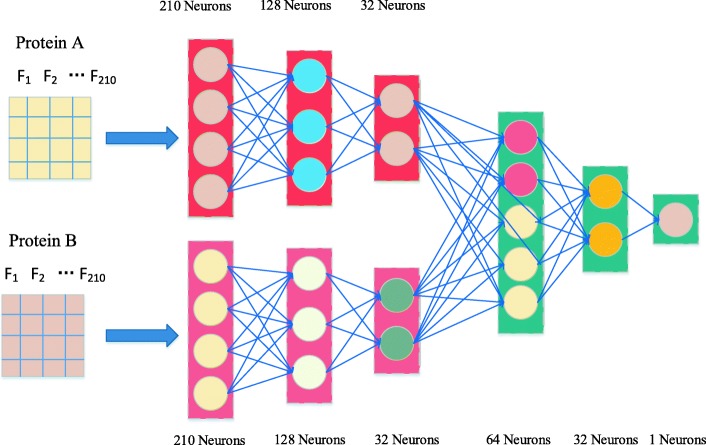
The framework of our deep neural networks for protein-protein interactions prediction

Deep neural networks (DNNs) are composed of an input layer, multiple hidden layers (three or more hidden layers), and an output layer, the configuration of adopted DNNs is shown in Fig. 2. In general, neural networks are fed data from the input layer (*x*), then the output values are sequentially computed along with hidden layers by transforming input data in a nonlinear way. Neurons of a hidden layer or output layer are connected to all neurons of the previous layer [32]. Each neuron computes a weighted sum of its inputs and applies a nonlinear activation function to calculate its outputs *f*(*x*) [32]. The nonlinear activation functions usually include sigmoid, hyperbolic tangent, or rectified linear unit (ReLU) [57]. ReLU and sigmoid are employed inthis work.

We separately construct two DNNs using TensorFlow platform, as illustrated in Fig. 2. Next, the feature vectors of two individual proteins extracted by AC are employed as the inputs for these two DNNs, respectively. After that, these two separate DNNs were combined in a hidden layer to predict PPIs. Adam algorithm [58] (an adaptive learning rate methods) is applied to speed up training. Meanwhile, the dropout technique is employed to avoid overfitting. The ReLU activation function [57] and cross entropy loss are employed, since they can both accelerate the model training and obtain better prediction results [59]. The batch normalization approach is also applied to reduce the dependency of training with the parameter initialization, speed up training and minimize the risk of overfitting. The following equations are used to calculate the loss:

5 $$ \begin{aligned} \mathbf{H}_{i1}^m= \sigma_{1}(\mathbf{W}_{i1}\mathbf{X}_{i1}+\mathbf{b}_{i1}) (i=1,\cdots,n; m = 1,2) \\ \end{aligned}  $$

6 $$ \begin{aligned} \mathbf{H}_{ij}^m= \sigma_{1}(\mathbf{W}_{ij}\mathbf{H}_{i(j-1)}+\mathbf{b}_{ij}) \\ (i=1,\cdots,n; j=2,\cdots,h_{1}; m=1,2)\\ \end{aligned}  $$

7 $$ \begin{aligned} \mathbf{H}_{ik}^3= \sigma_{1}\left(\mathbf{W}_{ik}\left(\mathbf{H}_{{ih}_{1}}^{1} \oplus \mathbf{H}_{{ih}_{1}}^{2}\right) +\mathbf{b}_{ik}\right)\\ (i=1,\cdots,n, k=h_{1}+1) \\ \end{aligned}  $$

8 $$ \begin{aligned} \mathbf{H}_{ik}^3= \sigma_{1}\left(\mathbf{W}_{ik}\mathbf{H}_{i(k-1)}+\mathbf{b}_{ik}\right) \\ (i=1,\cdots,n; k=h_{1}+2,\cdots,h_{2})\\ \end{aligned}  $$

9 $$ \begin{aligned} L=&-\frac{1}{n}\sum_{i=1}^{n}[\!\mathbf{y}_{i}ln(\sigma_{2}(\mathbf{W}_{{ih}_{2}}\mathbf{H}_{{ih}_{2}}+\mathbf{b}_{{ih}_{2}})\\ &+(1-\mathbf{y}_{i})ln(1-\sigma_{2}(\mathbf{W}_{{ih}_{2}}\mathbf{H}_{{ih}_{2}}+\mathbf{b}_{{ih}_{2}}))] \end{aligned}  $$

where *n* is the number of PPIs for batch training, *m* represents the individual network, and *h*_1_ is the depth of two individual networks, *h*_2_ is the depth of fused network. *σ*_1_ is the activation function of ReLU, *σ*_2_ is the activation function of the output layer with sigmoid, ⊕ represents the concatenation operator. **X** is the batch training inputs, **H** is the output of hidden layer, and **y** is the corresponding desired outputs. **W** is the weight matrix between the input layer and output layer, **b** is the bias.

## Results and discussion

In this section, we briefly introduce several widely-used evaluation criteria for performance comparison, and the recommended configuration of experiments. Next, we analyze and discuss the experimental results and compare our results with those of other related work.

### Evaluation metrics

To comprehensively compare the performance, six evaluation metrics are employed, accuracy (ACC), precision (PE), sensitivity (SEN), specificity (SPE), matthews correlation coefficient (MCC), *F*_1_ score values, and area under the receiver operating characteristic curve (AUC). These metrics (except AUC) are computed as follows:

10 $$\begin{array}{*{20}l} &ACC = \frac{TP+TN}{TP+TN+FP+FN}  \end{array} $$

11 $$\begin{array}{*{20}l} &SEN = \frac{TP}{TP+FN} \end{array} $$

12 $$\begin{array}{*{20}l} &SPE = \frac{TN}{TN+FP} \end{array} $$

13 $$\begin{array}{*{20}l} &PE = \frac{TP}{TP+FP} \end{array} $$

14 $$\begin{array}{*{20}l} &MCC = \frac{TP \times TN - FP \times FN}{\sqrt{(TP+FP)(TP+FN)(TN+FP)(TN+FN)}} \end{array} $$

15 $$\begin{array}{*{20}l} &F_{1} = \frac{2TP}{2TP+FP+FN} \end{array} $$

where true positive (TP) stands for the number of true PPIs which are correctly predicted; false negative (FN) stands for the number of true PPIs which are incorrectly predicted as non-interacting pairs; false positive (FP) is the number of true non-interacting pairs which are predicted as interacting pairs; true negative (TN) represents the number of true non-interacting pairs which are correctly predicted. MCC is considered as the most robust metric of a binary classifier. MCC equal to 0 represents completely random prediction, whereas 1 means perfect prediction. *F*_1_ score is a harmonic average of precision and sensitivity, and a larger score indicates a better performance. Receiver operating characteristic (ROC) curve is also employed to assess the performance of prediction model. To summarize ROC curve in single quantity, the area under ROC curve (AUC) is used. AUC ranges from 0 to 1, the maximum value 1 stands for perfect prediction. For a random guess, the AUC value is close to 0.5.

### Experimental setup

Our approach is implemented on TensorFlow platform https://www.tensorflow.org. We firstly constructed the negative datasets using four different strategies. We then encoded the amino acid sequences from the datasets using auto covariance (AC) [24]. After that, we trained two separate neural networks with graphics processing unit (GPU) based on the feature sets encoded by AC. Finally, we fused these two networks to predict new PPIs. Deep learning algorithms contains a number of hyper-parameters, which may heavily impact the experimental results. The recommended hyper-parameters configuration of our proposed model is summarized in Table 3. As to the parameter specification of the comparing methods, we employed the grid search to obtain the optimal parameters, which are shown in Table 4. For Du et al. [27] work, they also provided with a similar hyper-parameters configuration with ours, which can be accessed via the reference [27]. All the experiments are carried out on a server with configuration: CentOS 7.3, 256GB RAM, and Intel Exon E5-2678 v3. Meanwhile, we used NVIDIA Corporation GK110BGL [Tesla K40c] to accelerate training of DNNs.

**Table 3:** Recommended parameters of our model

Name	Range	Recommend
Learning rate	1,0.1,0.001,0.002,0.003,0.0001	0.002
Batch size	32,64,128,256,512,1024,2056	1024,2056
Weight initialization	uniform, normal, lecun_uniform, glorot_normal, glorot_uniform	glorot_normal
Per-parameter adaptive learning rate	SGD, RMSprop, Adagrad, Adadelta, Adam, Adamax, Nadam	Adam
Activation function	relu, tanh, sigmoid, softmax, softplus	relu, sigmoid
Dropout rate	0.5, 0.6, 0.7	0.6
Depth	2, 3, 4, 5, 6, 7, 8,9	3
Width	16, 32, 64, 128, 256, 1024, 2048, 4096	128, 64, 32
GPU	Yes, No	Yes

**Table 4:** Optimal parameters of comparing methods

Method	Name	Parameters			
Guo’s work [21]	SVM+AC	C	*γ*		Kernel
		32768.0	0.074325444687670064		Poly
Yang’s work [22]	*k*NN+LD	n_neighbors	Weights	Algorithm	p
		3	Distance	Auto	1
Zhou’s work [23]	SVM+LD	C	*γ*		Kernel
		3.1748021	0.07432544468767006		rbf
You’s work [25]	RF+MCD	n_estimators	Max_features	Criterion	Bootstrap
		5000	Auto	Gini	True

#### Contribution of controlling degrees

**Fig. 3:**
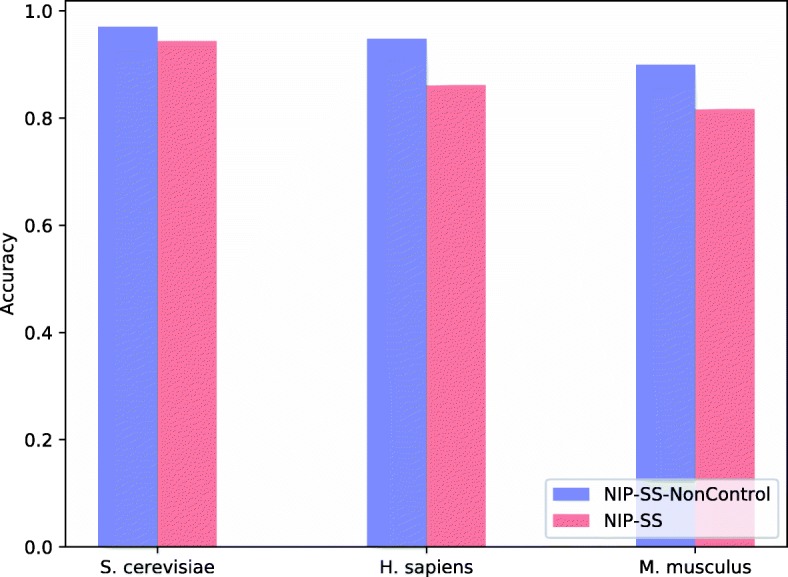
The experimental results of NIP-SS-NonControl and NIP-SS on *S. cerevisiae*, *H. sapiens*, and *M. musculus*. The negative datasets constructed by NIP-SS control the degree distribution of proteins

For the negative dataset generated by NIP-SS, we select the top-*m* protein-protein pairs with the lowest sequential similarity as the negative PPIs. Among all protein pairs, the similarity between these protein pairs is minimum. However, there are some proteins having very large degrees, which will lead to a bias and overestimation of prediction results. Therefore, we need to control the degree distribution of the negative dataset, and approximate the distribution with that of the positive dataset to guarantee the generalization ability of negative examples. Table 5 reports the degree distribution of proteins in *S. cerevisiae*, *H. sapiens* and *M. musculus*, and Fig. 3 reveals the prediction performance of NIP-SS with and without controlling the degree of proteins related to negative samples.

**Table 5:** The degree distribution of proteins of different datasets on *S. cerevisiae*, *H. sapiens*, and *M. musculus*

		*r*	Maximum degree	Number	The proportion of proteins/the number of interactions (non-interactions)								
					1<degree<10	10<degree<20	20<degree<30	30<degree<50	50<degree<70	70<degree<80	80<degree<100	100<degree<150	degree>150
*S. cerevisiae*	Positive	6.8763	252	4382	0.7976/0.3418	0.1130/0.2131	0.0436/0.1372	0.0299/0.1429	0.0087/0.0667	0.0014/0.0132	0.0030/0.0341	0.0025/0.0382	0.0005/0.0128
	NIP-SS-NonControl	8.0493	1439	3814	0.8589/0.2841	0.0679/0.1092	0.0260/0.0723	0.0207/0.0905	0.0105/0.0680	0.0029/0.0238	0.0026/0.0259	0.0042/0.0524	0.0063/0.2739
	NIP-SS	7.9391	154	3861	0.8052/0.3406	0.0873/0.1248	0.0303/0.0863	0.0544/0.2381	0.0080/0.0492	0.0049/0.0402	0.0028/0.0272	0.0065/0.0851	0.0005/0.0085
	Sub	12.7178	105	2516	0.6355/ 0.2302	0.2134/0.2196	0.0254/0.0467	0.0552/0.1928	0.0672/0.2902	0.0016/0.0089	0.0012/0.0085	0.0004/0.0031	0/0
	Random method	6.8781	18	4381	0.8274/0.7327	0.1726/0.2673	0/0	0/0	0/0	0/0	0/0	0/0	0/0
*H. sapiens*	Positive	1.8146	31	2384	0.9727/0.8470	0.0243/0.1262	0.0025/0.0219	0.0004/0.0049	0/0	0/0	0/0	0/0	0/0
	NIP-SS-NonControl	2.9263	312	1709	0.9427/0.4741	0.0234/0.0866	0.0164/0.1043	0.0053/0.0514	0.0070/0.1067	0.0018/0.0343	0/0	0.0029/0.0961	0.0006/0.0465
	NIP-SS	2.7760	30	1777	0.9572/0.8012	0.0315/0.1250	0.0113/0.0738	0/0	0/0	0/0	0/0	0/0	0/0
	Sub	6.5563	39	888	0.7331/0.3221	0.1486/0.2697	0.1002/0.3279	0.0180/0.0803	0/0	0/0	0/0	0/0	0/0
	Random method	2.0212	9	2221	1.0000/1.0000	0/0	0/0	0/0	0/0	0/0	0/0	0/0	0/0
*M. musculus*	Positive	0.9473	24	948	0.9926/0.9450	0.0063/0.0411	0.0011/0.0139	0/0	0/0	0/0	0/0	0/0	0/0
	NIP-SS-NonControl	1.9025	99	636	0.9513/0.5929	0.0299/0.1389	0.0110/0.0882	0.0016/0.0169	0.0016/0.0275	0.0016/0.0380	0.0031/0.0977	0/0	0/0
	NIP-SS	2.0163	20	612	0.9624/0.8212	0.0376/0.1788	0/0	0/0	0/0	0/0	0/0	0/0	0/0
	Sub	6.0190	52	263	0.7262/0.1446	0.1901/0.3857	0/0	0.0798/0.4415	0.0038/0.0282	0/0	0/0	0/0	0/0
	Random method	1.2678	8	814	1/1	0/0	0/0	0/0	0/0	0/0	0/0	0/0	0/0

From Table 5, we can see that the maximum degree of proteins in the negative dataset (NIP-SS-NonControl) is 1439, and the proportion of non-interactions with degree larger than 150 is 27.39%, which may lead to a bias. As a result, using this datasets produce a higher accuracy of 97.05%. Compared to NIP-SS-NonControl, the negative dataset constructed by NIP-SS contains more proteins and smaller maximum degree. Meanwhile, non-interactions are mainly related to proteins whose degrees fewer than 50. As such, the negative dataset generated by NIP-SS has a better generalization ability and lower bias than that by NIP-SS-NonControl. The contribution of controlling the degree of proteins in the negative dataset is also significant on *H. sapiens* and *M. musculus* datasets.

If we directly select protein pairs whose corresponding entries equal to 0 in the updated **W**^(*k*)^ to generate the negative dataset, such a dataset brings less predictive information and is not conducive for predicting PPIs, since this dataset contains many proteins with low degrees. Therefore, a sub-matrix **W**_*p*×*p*_ is employed to control the degree distribution of proteins. In addition, *k* also affects the degree distribution. Given that, we need to specify suitable input values of *p* and *k*. Particularly, we firstly fix *k* to 3, and then tune *p* from 500 to 4382 with an interval of 500. Next, we calculate the average repeatability (*r*), maximum degree of proteins, the proportion of proteins in different ranges of degrees, and the proportion of non-interactions in each range. We then choose *p* that makes the degree of proteins in the negative dataset similar to those of the positive dataset. After that, we adopt *p* selected in the first step and tune *k* within {1,2,3,6,10,50,300,1000}.

**Fig. 4:**
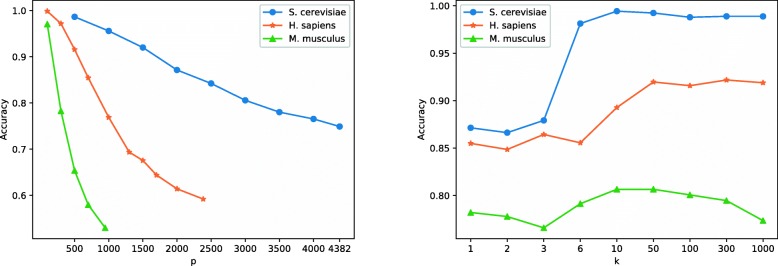
Left: the accuracy under different input values of *p* (the size of submatrix). Right: the accuracy under different input values of *k* (the steps of random walks)

The degree distribution and prediction results on *S. cerevisiae* are shown in Table 6 and Fig. 4, respectively. From Table 6, we can see that when *p*≈2000, the degree distribution of the negative dataset is most similar to that of the positive dataset. In addition, from Fig. 4, we can also observe that when *n*<2000, the accuracy with the setting *p*=500,1000 and *k*=3 is higher than 95%. This is because the average repeatability is large and leads to a bias.

**Table 6:** The degree distribution of proteins of different sizes of submatrix **W**_*p*×*p*_ (*k*=3) on *S. cerevisiae*, *H. sapiens*, and *M. musculus*

		*r*	Maximum degree	Number	The proportion of proteins/the number of interactions (non-interactions)								
					1<degree<10	10<degree<20	20<degree<30	30<degree<50	50<degree<70	70<degree<80	80<degree<100	100<degree<150	degree>150
*S. cerevisiae*	Positive	6.8763	252	4382	0.7976/0.3418	0.1130/0.2131	0.0436/0.1372	0.0299/0.1429	0.0087/0.0667	0.0014/0.0132	0.0030/0.0341	0.0025/0.0382	0.0005/0.0128
	*p*=500	35.6391	99	942	0.0106/0.0022	0.0998/0.0447	0.1943/0.1396	0.6008/0.6468	0.0669/0.1024	0.0096/0.0200	0.0180/0.0443	0/0	0/0
	*p*=1000	18.4008	56	1779	0.1417/0.0560	0.4817/0.3978	0.2816/0.3518	0.0877/0.1744	0.0073/0.0200	0/0	0/0	0/0	0/0
	*p*=1500	12.9057	48	2482	0.3429/0.1847	0.5028/0.5189	0.1241/0.2219	0.0302/0.0745	0/0	0/0	0/0	0/0	0/0
	*p*=2000	10.2938	36	3056	0.5301/0.3250	0.3750/0.4727	0.0906/0.1900	0.0043/0.0123	0/0	0/0	0/0	0/0	0/0
	*p*=2500	8.7113	29	3554	0.6294/0.4206	0.3348/0.4952	0.0357/0.0841	0/0	0/0	0/0	0/0	0/0	0/0
	*p*=3000	7.8181	28	3914	0.6704/0.4607	0.3143/0.5003	0.0153/0.0390	0/0	0/0	0/0	0/0	0/0	0/0
	*p*=3500	7.2907	24	4163	0.7173/0.5390	0.2782/0.4491	0.0046/0.0119	0/0	0/0	0/0	0/0	0/0	0/0
	*p*=4000	6.9820	24	4324	0.7590/0.6048	0.2396/0.3913	0.0014/0.0038	0/0	0/0	0/0	0/0	0/0	0/0
	*p*=4382	6.9070	24	4365	0.7830/0.6512	0.2160/0.3463	0.0009/0.0025	0/0	0/0	0/0	0/0	0/0	0/0
*H. sapiens*	Positive	1.8146	31	2384	0.9727/0.8470	0.0243/0.1262	0.0025/0.0219	0.0004/0.0049	0/0	0/0	0/0	0/0	0/0
	*p*=100	33.4103	73	195	0/0	0.0051/0.0030	0.2410/0.1938	0.7282/0.7548	0.0205/0.0376	0.0051/0.0109	0/0	0/0	0/0
	*p*=300	10.7926	28	569	0.4095/0.2923	0.5536/0.6335	0.0369/0.0742	0/0	0/0	0/0	0/0	0/0	0/0
	*p*=500	6.4638	25	899	0.8365/0.7101	0.1613/0.2829	0.0022/0.0070	0/0	0/0	0/0	0/0	0/0	0/0
	*p*=700	4.6913	18	1179	0.9245/0.8328	0.0755/0.1672	0/0	0/0	0/0	0/0	0/0	0/0	0/0
	*p*=1000	3.3799	18	1532	0.9778/0.9371	0.0222/0.0629	0/0	0/0	0/0	0/0	0/0	0/0	0/0
	*p*=1300	2.7423	14	1793	0.9933/0.9791	0.0067/0.0209	0/0	0/0	0/0	0/0	0/0	0/0	0/0
	*p*=1500	2.4481	13	1946	0.9964/0.9881	0.0036/0.0119	0/0	0/0	0/0	0/0	0/0	0/0	0/0
	*p*=1700	2.2924	11	2038	0.9995/0.9984	0.0005/0.0016	0/0	0/0	0/0	0/0	0/0	0/0	0/0
	*p*=2000	2.1370	10	2139	1/1	0/0	0/0	0/0	0/0	0/0	0/0	0/0	0/0
	*p*=2384	2.0171	10	2224	1/1	0/0	0/0	0/0	0/0	0/0	0/0	0/0	0/0
*M. musculus*	Positive	0.9473	24	948	0.9926/0.9450	0.0063/0.0411	0.0011/0.0139	0/0	0/0	0/0	0/0	0/0	0/0
	*p*=100	8.6649	22	191	0.6545/0.5287	0.3351/0.4480	0.0105/0.0233	0/0	0/0	0/0	0/0	0/0	0/0
	*p*=300	2.6700	13	503	0.9841/0.9474	0.0159/0.0526	0/0	0/0	0/0	0/0	0/0	0/0	0/0
	*p*=500	1.7843	10	663	1/1	0/0	0/0	0/0	0/0	0/0	0/0	0/0	0/0
	*p*=700	1.4745	10	746	1/1	0/0	0/0	0/0	0/0	0/0	0/0	0/0	0/0
	*p*=948	1.2214	7	831	1/1	0/0	0/0	0/0	0/0	0/0	0/0	0/0	0/0

The similar parameter selection strategy is also conducted on the other two datasets. The experimental results and the degree distribution of proteins are shown in Fig. 4, Tables 6, and 7. According to Table 6, we set *p*=700 and *p*=300 for *H. sapiens* and *M. musculus*, respectively. In addition, according to the right of Fig. 4 and Table 7, we fix *k*=3 for *H. sapiens* and *k*=50 for *M. musculus*. From Table 6, we can also observe that when *p* is set to *n*/2 (or *n*/3, *n* is the number of proteins in the positive set), the degree distribution generally approximates well with that of the positive dataset.

**Table 7:** The degree distribution of proteins under different *k* on *S. cerevisiae*, *H. sapiens*, and *M. musculus*

			*r*	Maximum degree	Number	The proportion of proteins/the number of interactions (non-interactions)								
						1<degree<10	10<degree<20	20<degree<30	30<degree<50	50<degree<70	70<degree<80	80<degree<100	100<degree<150	degree>150
*S. cerevisiae*	Positive		6.8763	252	4382	0.7976/0.3418	0.1130/0.2131	0.0436/0.1372	0.0299/0.1429	0.0087/0.0667	0.0014/0.0132	0.0030/0.0341	0.0025/0.0382	0.0005/0.0128
	*p*=2000	*k*=1	10.1949	33	3083	0.5336/0.3501	0.4032/0.5196	0.0626/0.1285	0.0006/0.0019	0/0	0/0	0/0	0/0	0/0
		*k*=2	10.2095	22	3079	0.5320/0.3452	0.4005/0.5138	0.0659/0.1364	0.0016/0.0046	0/0	0/0	0/0	0/0	0/0
		*k*=3	10.2131	39	3078	0.5338/0.3312	0.3765/0.4765	0.0854/0.1800	0.0042/0.0123	0/0	0/0	0/0	0/0	0/0
		*k*=6	10.2460	583	3069	0.8661/0.3685	0.0909/0.1090	0.0130/0.0292	0.0075/0.0257	0.0033/0.0169	0.0013/0.0089	0.0016/0.0128	0.0010/0.0110	0.0153/0.4179
		*k*=10	10.1841	679	3086	0.9180/0.4234	0.0680/0.0731	0/0	0/0	0/0	0/0	0/0	0/0	0.0139/0.5035
		*k*=50	10.3570	658	3039	0.9105/0.4172	0.0760/0.0797	0/0	0/0	0/0	0/0	0/0	0/0	0.0135/0.5031
		*k*=100	10.2350	574	3072	0.9134/0.4201	0.0713/0.0760	0/0	0/0	0/0	0/0	0/0	0/0	0.0153/0.5038
		*k*=300	10.2754	598	3061	0.9138/0.4205	0.0699/0.0749	0.0007/0.0012	0/0	0/0	0/0	0/0	0/0	0.0157/0.5034
		*k*=1000	10.2865	642	3058	0.9094/0.4156	0.0755/0.0808	0/0	0/0	0/0	0/0	0/0	0/0	0.0150/0.5036
*H. sapiens*	Positive		1.8146	31	2384	0.9727/0.8470	0.0243/0.1262	0.0025/0.0219	0.0004/0.0049	0/0	0/0	0/0	0/0	0/0
	*p*=700	*k*=1	4.5824	21	1202	0.9085/0.8057	0.0907/0.1914	0.0008/0.0029	0/0	0/0	0/0	0/0	0/0	0/0
		*k*=2	4.6720	18	1183	0.9231/0.8282	0.0769/0.1718	0/0	0/0	0/0	0/0	0/0	0/0	0/0
		*k*=3	4.6339	23	1191	0.9278/0.8351	0.0714/0.1614	0.0008/0.0034	0/0	0/0	0/0	0/0	0/0	0/0
		*k*=6	4.6577	19	1186	0.9115/0.8004	0.0885/0.1996	0/0	0/0	0/0	0/0	0/0	0/0	0/0
		*k*=10	4.8196	26	1153	0.8803/0.6975	0.1110/0.2686	0.0087/0.0340	0/0	0/0	0/0	0/0	0/0	0/0
		*k*=50	4.7895	29	1159	0.8645/0.6432	0.1182/0.2875	0.0173/0.0693	0/0	0/0	0/0	0/0	0/0	0/0
		*k*=100	4.9486	31	1128	0.8608/0.6405	0.1232/0.2942	0.0151/0.0607	0.0009/0.0046	0/0	0/0	0/0	0/0	0/0
		*k*=300	4.9119	28	1135	0.8520/0.6191	0.1251/0.2912	0.0229/0.0897	0/0	0/0	0/0	0/0	0/0	0/0
		*k*=1000	4.8348	29	1150	0.8678/0.6590	0.1165/0.2766	0.0157/0.0644	0/0	0/0	0/0	0/0	0/0	0/0
*M. musculus*	Positive		0.9473	24	948	0.9926/0.9450	0.0063/0.0411	0.0011/0.0139	0/0	0/0	0/0	0/0	0/0	0/0
	*p*=300	*k*=1	2.8863	14	475	0.9916/0.9745	0.0084/0.0255	0/0	0/0	0/0	0/0	0/0	0/0	0/0
		*k*=2	2.7293	13	495	0.9879/0.9615	0.0121/0.0385	0/0	0/0	0/0	0/0	0/0	0/0	0/0
		*k*=3	2.8140	14	484	0.9897/0.9670	0.0103/0.0330	0/0	0/0	0/0	0/0	0/0	0/0	0/0
		*k*=6	2.8062	12	485	0.9959/0.9875	0.0041/0.0125	0/0	0/0	0/0	0/0	0/0	0/0	0/0
		*k*=10	2.7673	13	490	0.9939/0.9794	0.0061/0.0206	0/0	0/0	0/0	0/0	0/0	0/0	0/0
		*k*=50	2.8062	14	485	0.9876/0.9599	0.0124/0.0401	0/0	0/0	0/0	0/0	0/0	0/0	0/0
		*k*=100	2.8219	14	483	0.9834/0.9502	0.0166/0.0498	0/0	0/0	0/0	0/0	0/0	0/0	0/0
		*k*=300	2.8863	15	475	0.9789/0.9350	0.0211/0.0650	0/0	0/0	0/0	0/0	0/0	0/0	0/0
		*k*=1000	2.7906	15	487	0.9856/0.9529	0.0144/0.0471	0/0	0/0	0/0	0/0	0/0	0/0	0/0

### Results of different negative dataset construction strategies

To investigate the effectiveness of the proposed two strategies for constructing negative dataset, we conduct experiments on three prevalent PPIs datasets, including *S. cerevisiae*, *H. sapiens* and *M. musculus* datasets, and take the performance of PPIs prediction as the comparing index. To avoid over-fitting and data dependency, five-fold cross-validation is adopted. Table 8 reports the average prediction results on these three species using different negative dataset generation strategies.

**Table 8:** Results based on different negative datasets on *S. cerevisiae*, *H. sapiens* and *M. musculus*

Species	Negative samples	ACC	PE	RE	SPE	MCC	*F*_1_	AUC
*S. cerevisiae*	NIP-SS	94.34% ± 0.38%	95.62% ± 0.75%	92.96% ± 0.40%	95.74% ± 0.75%	88.73% ± 0.77%	94.27% ± 0.34%	98.24% ± 0.11%
	NIP-RW	87.92% ± 0.24%	90.04% ± 1.69%	85.32% ± 1.90%	90.48% ± 2.20%	75.97% ± 0.55%	87.59% ± 0.35%	94.23% ± 0.12%
	Sub	93.79% ± 0.43%	95.18% ± 0.41%	92.25% ± 0.78%	95.33% ± 0.45%	87.62% ± 0.83%	93.69% ± 0.38%	98.13% ± 0.17%
	Random method	74.20% ± 0.78%	72.68% ± 1.45%	77.59% ± 0.89%	70.83% ± 1.77%	48.53% ± 1.47%	75.04% ± 0.76%	81.29% ± 0.34%
*H. sapiens*	NIP-SS	86.17% ± 0.93%	86.38% ± 1.27%	85.88% ± 1.55%	86.48% ± 1.00%	72.36% ± 1.85%	86.12% ± 1.05%	92.20% ± 0.82%
	NIP-RW	86.44% ± 0.59%	90.05% ± 0.48%	81.87% ± 2.35%	90.91% ± 1.09%	73.14% ± 1.12%	85.75% ± 1.28%	92.30% ± 0.70%
	Sub	93.34% ± 0.58%	93.19% ± 0.42%	93.51% ± 0.94%	93.17% ± 0.37%	86.68% ± 1.16%	93.35% ± 0.57%	96.22% ± 0.45%
	Random method	60.46% ± 1.54%	60.07% ± 1.74%	62.33% ± 1.91%	58.50% ± 3.24%	20.85% ± 3.14%	61.17% ± 1.63%	64.57% ± 1.35%
*M. musculus*	NIP-SS	81.69% ± 1.48%	80.57% ± 2.20%	83.73% ± 2.97%	79.51% ± 4.47%	63.44% ± 3.16%	82.06% ± 0.84%	87.04% ± 1.95%
	NIP-RW	80.66% ± 2.14%	84.89% ± 5.41%	74.83% ± 3.46%	86.72% ± 4.62%	61.97% ± 4.53%	79.41% ± 2.52%	87.75% ± 2.25%
	Sub	91.82% ± 1.26%	90.13% ± 2.57%	93.93% ± 2.38%	89.76% ± 2.41%	83.78% ± 2.40%	91.95% ± 1.44%	94.81% ± 0.74%
	Random method	50.76% ± 2.12%	50.80% ± 5.77%	52.17% ± 1.90%	49.44% ± 3.26%	1.60% ± 3.86%	51.37% ± 3.58%	51.40% ± 2.43%

We can see that for the *S. cerevisiae* dataset, the model based on the negative dataset generated by NIP-SS gives the average accuracy of 94.34%, precision of 95.62%, recall of 92.96%, specificity of 95.74%, MCC of 88.73%, *F*_1_ of 94.27% and AUC of 98.24%, respectively. These values are higher than those of other strategies, which separately adopt random walk, random pairing, subcellular localization to generate the negative dataset. These results prove the effectiveness of NIP-SS in generating reliable non-interacting protein pairs for PPIs prediction. In addition, the negative dataset constructed by NIP-SS contain more proteins and have similar degree distributions to the positive dataset, which can effectively control the bias of the dataset. The model trained on the negative dataset generated by random pairing yields very low accuracy of 74.20%. That is because this negative dataset has a high rate of false negatives, and the degree distribution mainly concentrates on proteins with degree smaller than 10. The model based on negative dataset generated by subcellular localization also yields a good performance with accuracy of 93.79%, MCC of 87.62%, and AUC of 98.13%. However, compared to the negative dataset generated by NIP-SS, this dataset covers fewer proteins and a larger proportion of non-interactions in the degree range 50-70, which are higher than those NIP-SS. Those will produce an over-optimistic estimate of prediction.

The model trained on the negative dataset generated by NIP-RW yields an average accuracy of 87.92%, MCC of 75.97% and AUC of 94.23%. These values are lower than those of NIP-SS. That is mainly because the proteins in the negative datasets generated by NIP-SS and NIP-RW have different degrees. 21.05% non-interacting protein pairs in the negative dataset generated by NIP-SS are located in range of degree larger than 50, but no non-interacting protein pairs in the negative dataset generated by NIP-RW are located in that range. Another reason is that random walk process is restricted by the connected positive examples. For the small network of *H. sapiens* and *M. musculus* datasets, NIP-RW yields good results.

As to the *H. sapiens* and *M. musculus* datasets, we can observe that the model based on the negative datasets of subcellular localization yields the best prediction accuracy of 93.34% and 91.82%, respectively. We find the negative datasets constructed by subcellular localization has the maximum average repeatability (*r*) and contains the fewest proteins, which lead to a bias and an overestimated performance. Since the degree distribution of negative datasets constructed by NIP-SS and NIP-RW are similar, the prediction performance using these two strategies are similar. The model based on negative datasets generated by random pairing again gives the lowest performance.

To further investigate the effectiveness of our model that uses two separate DNNs at first, we introduced a variant of our model called DNNs-Con. DNNs-Con firstly concatenates AC features of two individual proteins, and then takes the concatenated features as input of DNNs. The hidden layers for this network are fixed as 420-256-32. To check the statistical significance between our model and DNNs-Con, the pairwise *t*-test (at 95% significance level) is also used. The experimental results of five-fold cross validation are reported in Table 9. From Table 9, we can observe that the accuracy, MCC, *F*_1_ and AUC of our model are 2.61%, 5.22%, 2.68% and 1.29% higher than those of DNNs-Con, respectively. In addition, we observe that our model converges faster than DNNs-Con during the training process, that is due to two separate networks can faster extract sequence information contained in each amino acid sequence. These results prove that our model (using two separate DNNs, instead of single one) is efficient and effective to predict PPIs.

**Table 9:** Results of different network architectures on *S. cerevisiae*, the adopted negative dataset is constructed by NIP-SS

Architectures	Data set	ACC	PE	RE	SPE	MCC	*F*_1_	AUC
DNNs	Fold 1	94.08%	94.04%	94.17%	93.98%	88.15%	94.11%	98.24%
	Fold 2	94.03%	94.36%	93.64%	94.42%	88.07%	94.00%	98.13%
	Fold 3	94.57%	95.25%	93.66%	95.45%	89.14%	94.45%	98.17%
	Fold 4	94.38%	94.99%	93.78%	94.98%	88.77%	94.38%	98.16%
	Fold 5	94.19%	94.84%	93.50%	94.88%	88.39%	94.17%	98.03%
	Average	94.25% ± 0.22%	94.70% ± 0.49%	93.75% ± 0.26%	94.74% ± 0.56%	88.5% ± 0.45%	94.22% ± 0.19%	98.15% ± 0.08%
DNNs-Con	Fold 1	91.92%	92.40%	91.27%	92.55%	83.84%	91.83%	97.15%
	Fold 2	91.86%	93.87%	89.21%	94.40%	83.79%	91.48%	96.90%
	Fold 3	91.58%	93.62%	89.32%	93.86%	83.26%	91.42%	96.83%
	Fold 4	91.86%	93.65%	90.07%	93.70%	83.79%	91.83%	96.92%
	Fold 5	91.42%	92.24%	90.53%	92.32%	82.86%	91.38%	96.93%
	Average	91.73% ± 0.21% ∙	93.16% ± 0.77% ∙	90.08% ± 0.86% ∙	93.37% ± 0.89% ∙	83.51% ± 0.43% ∙	91.59% ± 0.23% ∙	96.95% ± 0.12% ∙

∙/∘ indicates whether our model is statistically (according to pairwise *t*-test at 95% significance level) superior/inferior to the DNNs-Con

Based on the above analysis, we fix *p*=2000 and vary *k*∈{1,2,3,6,10,50,300,1000}. Figure 4 (right of this Figure) reports the results under different values of *k*. We also calculate the degree distribution at different *k*, which are listed in Table 7. From the right of Fig. 4, we can observe that when *k*≥6, the result is close to 1. That is because there are more nonzero entries in **W**^(*k*)^ as *k* increases, which change the degree distribution of proteins and thus bring in a larger bias. Table 7 shows the degree distribution when *p*=2000. Based on these results, we fix *k* to 3.

### The impact of of imbalanced class

In general, the number of negative PPIs has a large impact on prediction performance. To investigate the impact of imbalanced class on our proposed two strategies, three *H. sapiens* datasets are constructed with different numbers of negative samples for NIP-SS and NIP-RW, respectively. The ratios of positive samples (3355 interaction pairs) and negative samples in these three datasets are 1:1, 1:2 and 1:3, respectively. Four metrics of sensitivity (SEN), specificity (SPE), area under the receiver operating characteristic curve (AUC), and geometric mean (GM) are used to evaluate the prediction performance. GM is commonly used for class-imbalance learning [60], it can give a more accurate evaluation on imbalanced data. The GM is calculated by this formula: $GM=\sqrt {SEN \times SPE}$. The prediction results are shown in Table 10. From the Table 10, we can see that as the number of negative samples increases, the overall performance of the model shows a downward trend. In addition, the prediction values of AUC and GM decrease significantly. AUC is respectively decreased by 10.51% and 8.27% for NIP-SS and NIP-RW, and GM is decreased by 16.63% and 11.27%. Given that, to avoid the performance degradation caused by imbalanced class, we adopt the widely-used solution that uses the same number of negative PPIs as that of positive samples.

**Table 10:** Results on H. sapiens with different numbers of negative samples for NIP-SS and NIP-RW

Method	Dataset	SEN	SPE	AUC	GM
NIP-SS	*H*.*sapiens*_1:1_	86.57%	87.08%	92.01%	86.57%
	*H*.*sapiens*_1:2_	69.95%	89.25%	86.33%	79.00%
	*H*.*sapiens*_1:3_	52.93%	92.48%	81.50%	69.94%
NIP-RW	*H*.*sapiens*_1:1_	81.87%	90.91%	92.30%	86.27%
	*H*.*sapiens*_1:2_	72.84%	90.13%	87.41%	81.02%
	*H*.*sapiens*_1:3_	58.33%	94.51%	84.03%	75.00%

### The impact of different feature descriptors

The extracted features can affect the performance of PPIs prediction [28]. To investigate the contribution of auto covariance (AC) [21] descriptor, we separately train DNNs on *S. cerevisiae* (the negative dataset constructed by NIP-SS) based on AC [21], CT [20], LD [23], and MCD [25]. Table 11 reports the results of five-fold cross validation. Meanwhile, we also use pairwise *t*-test (at 95% significance level) to check the statistical significance between AC and CT, LD, MCD. From Table 11, we observe that DNNs-AC achieves an average accuracy as 94.25%, precision as 94.7%, recall as 93.75%, specificity as 94.74%, MCC as 88.5%, *F*_1_ as 94.22%, and AUC as 98.15%. The performance difference of these descriptors is not significant, but AC descriptors have the smallest feature dimension. For this reason, we adopt AC to encode amino acid sequences.

**Table 11:** Results of DNNs with AC, CT, LD and MCD feature descriptors on *S. cerevisiae*

Model	Dimension	ACC	PE	RE	SPE	MCC	*F*_1_	AUC
DNNs-AC	(210+210)	94.25% ± 0.22%	94.70% ± 0.49%	93.75% ± 0.26%	94.74% ± 0.56%	88.50% ± 0.45%	94.22% ± 0.19%	98.15% ± 0.08%
DNNs-CT	(343+343)	94.37% ± 0.24%	95.55% ± 0.75%	93.09% ± 0.81%	95.67% ± 0.65%	88.78% ± 0.48%	94.30% ± 0.23%	98.20% ± 0.21%
DNNs-LD	(630+630)	94.41% ± 0.14%	95.46% ± 0.41%	93.25% ± 0.44%	95.56% ± 0.44%	88.84% ± 0.28%	94.34% ± 0.15%	98.23% ± 0.06%
DNNs-MCD	(882+882)	94.25% ± 0.22%	94.70% ± 0.49%	93.75% ± 0.26%	94.74% ± 0.56%	88.50% ± 0.45%	94.22% ± 0.19%	98.15% ± 0.08%

∙/∘ indicates whether DNNs-AC is statistically (according to pairwise *t*-test at 95% significance level) superior/inferior to the other descriptors

### Comparison with existing methods

To further study the performance of our model and the contribution of negative dataset generated by NIP-SS and NIP-RW, we compare our prediction results on *S. cerevisiae* with those of other competitive methods, including Guo et al. [21], Yang et al. [22], Zhou et al. [23], You et al. [25], and Du et al. [27]. These approaches were introduced in “Background” section.

Table 12 shows the experimental results. Our method yields average prediction accuracy of 94.34%, precision of 95.62%, recall of 92.96%, MCC of 88.73%, *F*_1_ of 94.27%, and AUC of 98.24%. Compared to the other two negative datasets, the negative dataset constructed by NIP-SS covers more proteins and the degree distribution is close to the degree distribution of the positive dataset. In addition, we can observe that the comparing methods using the negative dataset constructed by NIP-RW also produces good results. However, for a large datasest, the degrees of proteins in the negative dataset generated by NIP-RW are almost always smaller than 50. This is because the distribution of degree is restricted by the collected positive examples and a large network makes the random walk process less controlled. For this reason, the NIP-RW is reliable on *H. sapiens* and *M. musculus*. These results prove that the negative datasets constructed by NIP-SS and NIP-RW are rational and can boost the performance of PPI prediction.

**Table 12:** Results of our modal and of other state-of-the-art methods on *S. cerevisiae*

Method	Negative samples	ACC	PE	RE	SPE	MCC	F1	AUC
Our method	Sub	93.79% ± 0.43%	95.18% ± 0.41%	92.25% ± 0.78%	95.33% ± 0.45%	87.62% ± 0.83%	93.69% ± 0.38%	98.13% ± 0.17%
	NIP-SS	94.34% ± 0.38%	95.62% ± 0.75%	92.96% ± 0.40%	95.74% ± 0.75%	88.73% ± 0.77%	94.27% ± 0.34%	98.24% ± 0.11%
	NIP-RW	87.92% ± 0.24%	90.04% ± 1.69%	85.32% ± 1.90%	90.48% ± 2.20%	75.97% ± 0.55%	87.59% ± 0.35%	94.23% ± 0.12%
Du’s work [27]	Sub	92.58% ± 0.38%	94.21% ± 0.45%	90.95% ± 0.41%	94.41% ± 0.45%	85.41% ± 0.76%	92.55% ± 0.39%	97.55% ± 0.16%
	NIP-SS	94.44% ± 0.35%	95.46% ± 0.38%	93.44% ± 0.45%	95.45% ± 0.41%	88.90% ± 0.68%	94.44% ± 0.37%	98.22% ± 0.20%
	NIP-RW	88.59% ± 0.32%	92.61% ± 0.41%	84.14% ± 0.43%	93.13% ± 0.35%	77.52% ± 0.59%	88.17% ± 0.34%	94.73% ± 0.18%
You’s work [25]	Sub	89.15% ± 0.33%	90.00% ± 0.57%	88.10% ± 0.17%	90.21% ± 0.61%	78.33% ± 0.67%	89.04% ± 0.31%	94.78% ± 0.21%
	NIP-SS	94.42% ± 0.47%	96.71% ± 0.47%	91.96% ± 0.64%	96.87% ± 0.46%	88.94% ± 0.92%	94.28% ± 0.49%	98.46% ± 0.12%
	NIP-RW	86.03% ± 0.43%	89.19% ± 0.60%	82.00% ± 0.70%	90.06% ± 0.64%	72.30% ± 0.85%	85.44% ± 0.46%	93.33% ± 0.46%
Zhou’s work [23]	Sub	88.76% ± 0.37%	89.44% ± 0.27%	87.89% ± 0.45%	89.62% ± 0.30%	77.53% ± 0.53%	88.66% ± 0.28%	94.69% ± 0.31%
	NIP-SS	92.10% ± 0.34%	93.48% ± 0.45%	90.51% ± 0.73%	93.68% ± 0.49%	84.24% ± 0.67%	91.97% ± 0.37%	97.29% ± 0.16%
	NIP-RW	82.64% ± 0.33%	83.98% ± 0.34%	80.67% ± 0.48%	84.61% ± 0.36%	65.34% ± 0.65%	82.30% ± 0.35%	90.00% ± 0.39%
Yang’s work [22]	Sub	84.81% ± 0.37%	87.53% ± 0.14%	81.18% ± 0.84%	88.44% ± 0.18%	69.80% ± 0.71%	84.23% ± 0.47%	90.03% ± 0.31%
	NIP-SS	89.18% ± 0.35%	93.34% ± 0.33%	84.38% ± 0.53%	93.98% ± 0.31%	78.73% ± 0.69%	88.64% ± 0.38%	95.50% ± 0.20%
	NIP-RW	83.98% ± 0.48%	86.09% ± 0.67%	81.07% ± 0.80%	86.89% ± 0.76%	68.09% ± 0.97%	83.50% ± 0.51%	91.45% ± 0.27%
Guo’s work [21]	Sub	87.88% ± 0.56%	88.16% ± 0.90%	87.53% ± 0.59%	88.24% ± 1.02%	75.77% ± 1.12%	87.84% ± 0.53%	93.69% ± 0.33%
	NIP-SS	90.00% ± 0.43%	90.45% ± 0.68%	89.45% ± 0.69%	90.55% ± 0.77%	80.01% ± 0.86%	89.94% ± 0.43%	95.02% ± 0.27%
	NIP-RW	82.43% ± 0.27%	83.48% ± 0.40%	80.87% ± 0.45%	83.99% ± 0.49%	64.89% ± 0.54%	82.15% ± 0.27%	89.04% ± 0.33%

### Results on independent datasets

Six independent datasets, which just only contain the examples of interactions (non-interactions), including *Caenorhabditis elegans* (4013 interacting pairs), *Escherichia coli* (6954 interacting pairs), *Helicobacter pylori* (1420 interact-ing pairs), *Homo sapiens* (1412 interacting pairs), *Mus musculus* (313 interacting pairs), and *Mammalian* (1937 non-interacting pairs), are employed as test sets to evaluate the generalization ability, and to further assess the practical prediction ability of our model and the rationality of NIP-SS and NIP-RW. Three datasets of *H. sapiens* (3355 positive examples and 3355 negative examples) are constructed and the difference between these datasets is the negative samples, which are generated by NIP-SS, NIP-RW, and subcellular location, respectively. Then, three models with optimal configuration (provided in “Evaluation metrics” section) are trained on these three datasets. After that, these six independent datasets are used to test the generalization ability of these models. The prediction results are shown in Table 13. From Table 13, we can observe that the accuracy of our model using the negative datasets generated by NIP-SS and NIP-RW on *C. elegans*, *E. coli*, *H. sapiens*, *H. pylori*, *M. musculus*, and *Mammalian* are 86.10%, 85.34%, 86.20%, 81.86%, 85.64%, 15.69% and 78.113%, 79.65%, 85.03%, 79.15%, 80.66%, 18.58%, respectively. These prediction results indicate that the negative datasets generated by NIP-SS and NIP-RW contribute to a good performance across species. We note that the accuracy on *Mammalian* using the NIP-SS and NIP-RW strategies are 3.36 and 3.98 times higher than that using subcellular localization (which is only 4.67%). Given that, we can conclude that the negative dataset generated by subcellular localization may produce a bias for predicting PPIs. In other words, subcellular localization based negative examples generation strategy is inclined to predict a new protein pair as interaction.To further demonstrate this discovery and the advantages of NIP-SS and NIP-RW, we constructed a dataset (named *Mammalian-imbalanced*), in which the number of negative samples is about 4 times than that of positive samples, since the number of protein pairs (non-interacting) is far greater than the number of interaction pairs in the real world. The negative samples are from *Mammalian* dataset (1937 negative samples), while the positive are from the *M. musculus* (313 positive samples). Finally, the dataset contains 313 + 1937 protein pairs. The prediction results are also shown in Table 13. From Table 13, we can see that the accuracy on *Mammalian-imbalanced* dataset using the NIP-SS and NIP-RW strategies are 23.45% and 27.56%, respectively, which are both higher than that using subcellular localization (only 17.75%). These prediction results show that NIP-SS and NIP-RW hold a good generalization ability and performance in predicting PPIs, and the strategies of subcellular location will lead to a bias in predicting.

**Table 13:** Prediction results on seven independent PPIs datasets, PPIs of *H. sapiens* are used as the training set

Species	Test pairs	Negative		
		NIP-SS	NIP-RW	Sub
*C. elegans*	4013 (interactions)	86.10%	78.11%	94.42%
*E. coli*	6984 (interactions)	85.34%	79.65%	92.68%
*H. sapiens*	1412 (interactions)	86.20%	85.03%	96.29%
*H. pylori*	1420 (interactions)	81.86%	79.15%	92.28%
*M. musculus*	313 (interactions)	85.64%	80.66%	96.10%
*Mammalian*	1937 (non-interactions)	15.69%	18.58%	4.67%
*Mammalian-imbalanced*	2250 (313 interactions, 1937 non-interactions)	23.45%	27.56%	17.75%

## Conclusion and future work

Effective PPIs prediction approaches depend on a high quality negative dataset (non-interacting protein pairs), which contributes to discriminative and accurate prediction. In this paper, we present two novel strategies (NIP-SS and NIP-RW) to generate high-quality negative dataset and to boost the performance of PPIs prediction. NIP-SS uses sequence similarity between proteins to guide the generation of negative examples, whereas NIP-RW utilizes the interaction profiles of proteins to select negative examples. To reduce the bias and enhance the generalization ability of the generated negative dataset, these two strategies separately adjust the degree of the non-interacting proteins and approximate the degree to that of the positive dataset. We found that NIP-SS is competent on all datasets and hold a good performance, whereas NIP-RW can only obtain a good performance on small dataset (positive samples ≤ 6000) because of the restriction of random walk and the results of extensive experiments. In addition, these experiments also indicate that the negative datasets constructed by NIP-SS and NIP-RW can significantly improve the performance of PPIs prediction and these two strategies work better than other two widely adopted strategies.

We will fuse multiple types of biological data, including the sequence similarity, functional similarity and domain similarity of proteins, to generate the negative datasets. In addition, we will investigate more intelligent ways to adjust the degree of non-interacting proteins.
